# Biological activities of sweet potato (*Ipomoea batatas* L.) tips and tubers

**DOI:** 10.1002/fsn3.2999

**Published:** 2022-07-26

**Authors:** Chae Young Hong, Yeon Jae Jo, Min Young Kim, Mi Nam Chung, Ehn‐Kyoung Choi, Yun‐Bae Kim, Junsoo Lee, Heon Sang Jeong

**Affiliations:** ^1^ Department of Food Science and Biotechnology Chungbuk National University Cheongju Korea; ^2^ National Institute of Crop Science Rural Development Administration Miryang Korea; ^3^ Bioenergy Crop Research Institute Rural Development Administration Muan Korea; ^4^ College of Veterinary Medicine Chungbuk National University Cheongju Korea

**Keywords:** antioxidant, biological activities, sweet potato tips, sweet potato tubers

## Abstract

This study was conducted to evaluate the biological activities of sweet potato tips and tubers. Antioxidant activity of 2,2‐azino‐bis 93‐ethlbenzothiazoline‐6‐sulphonic acid) diammonium salt (ABTS) and 1,1‐diphenyl‐2‐picrylhydrazyl (DPPH) radical scavenging activities had the highest value of 32.45 mg, AAE/g, and 15.10 mg AAE/g, respectively, in ‘*Pungwonmi*’ tips. Angiotensin converting enzyme I inhibitory activity ranged between 47.72% in ‘*Sinjami*’ tubers and 62.25% in ‘*Pungwonmi*’ tips. α‐Glucosidase inhibitory activity had the highest value of 78.81% and 62.93% in ‘*Pungwonmi*’ tips and ‘*Juhwangmi*’ tubers, respectively. In particular, ‘*Pungwonmi*’ tips had the most effective inhibiting effect on intracellular reactive oxygen species levels in HepG2 cells. Wound healing assay result revealed that ‘*Sinjami*’ showed 75% wound healing effect. For skin whitening, ‘*Pungwonmi*’ tips showed 63% activity at 10 mg/ml. These results suggest that sweet potato tips and tubers can be used to develop functional food and cosmetic materials.

## Graphical abstract

This study analyzed the biological activities of anti‐hypertension, anti‐diabetes, hepatoprotective, antioxidant, eye function, and skin whitening effects of sweet potato tips and tubers. Sweet potato tips and tubers will be used as functional food and cosmetic materials. Especially sweet potato tips will be excellent in improving eye disease, have whitening effects, and can be used as functional food and cosmetic material.

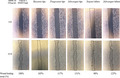

## 1 INTRODUCTION

Sweet potato (*Ipomoea batatas* L.) is an edible dicotyledon crop that belongs to the Convolvulaceae family. It is an excellent source of carbohydrates and a major food resource in Korea, along with rice and soybean. It is widely used as a functional food owing to its high content of not only carbohydrates but also phenolic compounds, carotenoids, vitamin C, anthocyanins, various minerals, and dietary fiber, and it is the 7th largest crop in the world (Bovell‐Benjamin, 2007). Sweet potato has attracted a lot of attention because it is a crop that has simultaneously solved energy, food, and environmental problems of the 21st century owing to its strong environmental adaptability, and its roots, stems, and leaves can be consumed (Ishida et al., 2000). In addition, sweet potato is a food with high utility value because it is used for producing food, snacks, processed foods, vegetables, industrial products, and feed (O'Hair, 1984).

The shape of sweet potato is largely divided into aboveground and underground parts. The aboveground part is composed of leaves, stems, flowers, and seeds, while the underground part is composed of roots. Sweet potato tips, which are 10 to 15 cm from the end of the stem, and leaves and stalks, which are among the shoots, are grown 50 to 60 days after tubers are directly cut into the field. Sweet potato tips can be harvested 8–10 times during the cultivation period, and the total yield is very high (130–180 t/ha) and has the advantage of easy harvesting (Lee, Park, et al., 2007). In recent years, the whole plant, tubers, tips, and stems of sweet potato are edible. Owing to the high content of polyphenols in the tips, it is used in the manufacturing of various foods in foreign countries by adding the dried powdered tips to dough for bread, ice cream, juice, and tea (Islam, 2006). In Korea, the leaves and stalks of sweet potato have been used as herbs or for soups for a long time, however, the tips are often discarded or used only as feed owing to its insufficient availability (Li et al., 2012). Sweet potato tips are not only rich in water‐soluble dietary fiber but also rich in protein compared to other vegetables. They are also rich in iron; potassium; vitamin B2, C, and E; carotene (Ishida et al., 2000); phenolic compounds; anthocyanins; and caffeic acid. They are known to also contain many antioxidants, including phosphoric acid derivatives (Islam et al., 2003).

Sweet potatoes are edible crops with both leaves, stems, and roots, and are highly economical and productive. In particular, sweet potato leaves and stems are known to have better antioxidant and phenolic components than regular vegetables. However, leaves and tips other than stems are treated as byproducts and are discarded or used as livestock feed. Therefore, in this study, functional ingredients and physiological activity were evaluated and their importance was verified in order to increase the availability of sweet potato tips and tubers and use them as various functional food materials.

## 2 MATERIALS AND METHODS

### 2.1 Preparation of sample

The sweet potato tips and tubers used in this experiment were harvested in 2019 at the Rural Development Administration's Bio Energy Center. Lutein‐high cultivars on *Juhwangmi* tips; β‐carotene‐high cultivar on *Hayanmi* tips, *Pungwonmi* tips, and Juhwangmi tubers, and anthocyanidin‐high cultivar on *Hayanmi* tips and *Sinjami* tubers were used. The harvested samples were washed with tap water, dried for 48 h in a 40°C hot air dryer (WFO‐459PD, EYELA, Tokyo, Japan), ground using a mill (Micro hammer cutter mill type‐3, Culatti AG, Zurich, Switzerland), and then stored at −4°C as a sample. Each sample was extracted with an 80% (v/v) methanol–water solution. Extraction was repeated three times for 1 h at 25°C using an ultrasonic extractor (frequency 40 Hz, power 300 W, SD‐350H; Seong Dong, Seoul, Korea). The extract was centrifuged at 2220× *g* (Centrifuge union 55 R, Hanil, Incheon, Korea) and then filtered (Advantec No. 2, Toyo Roshi Kaisha, Ltd, Tokyo, Japan).

### 2.2 ABTS and DPPH radical scavenging activity

ABTS and DPPH radical scavenging abilities were measured using the method described by Choi et al. (2006), with some modifications. After leaving the ABTS (7.4 mM) and potassium persulfate (2.6 mM) in the dark for one day to form ABTS. + cations so that the absorbance value is 1.4 at 735 nm, it was diluted with distilled water. To 1 ml of the diluted ABTS. + solution, 50 μl of the extract was dissolved in each concentration, and the change in absorbance was measured after exactly 30 min. The ABTS radical scavenging ability was expressed as the equivalent of ascorbic acid. DPPH radical scavenging activity was used after dissolving 0.00788 g of 0.2 mM DPPH in 99.9% ethanol and adjusted to 100 ml for 60 min. After adding 0.2 ml of the sample to 0.8 ml of the DPPH solution, the absorbance was measured at 520 nm by adding 0.2 ml of the sample at ambient temperature for 30 min, and the radical scavenging ability was expressed as an IC_50_ value.

### 2.3 Angiotensin converting enzyme I inhibitory activity

Angiotensin converting enzyme (ACE) inhibitory activity for each extract was measured using the method described by Kwon et al. (2006) with little modification. ACE inhibitory activity was measured using 5 mM HHL (Hippuryl‐His‐Leu) substrate and dissolved in 0.1 M potassium phosphate buffer (pH 8.3) containing 0.3 M NaCl, and the 0.2 mU ACE purifying enzyme was dissolved in 0.1 M potassium phosphate buffer (pH 7.0) and used. Eighty microliter of 0.2 mU ACE purified enzyme solution and 100 μl of 5 mM HHL substrate was added to 100 μl of the extract, and then left at 37°C for 60 min. To stop the reaction, 250 μl of 1 M HCl was added and then filtered through 0.45 μm syringe filters, and the resultant sample was then analyzed using a HPLC (ACME 9000 system, Younglin, Anyang, Korea), and the ACE inhibition rate (%) was calculated. The column was a C‐18 column (Mightysil RP‐18 GP column, 4.6 × 250 mm, Kanto Chemical, Tokyo, Japan), the mobile phase A was 10 mM phosphoric acid (pH 2.5), and the mobile phase B was a methanol. The initial ratio of A and B started at 100:0, followed by a stepwise gradient system with a ratio of 40:60 at 12 min, 0:100 at 19 min, and 100:0 at 25 min. The flow rate was 0.8 ml/min, the injection volume was 20 μl, and the UV detector (228 nm) was used for detection.

### 2.4 α‐Glucosidase inhibitory activity

α‐Glucosidase inhibitory activity was measured using the method described by Tibbot and Skadsen (1996). α‐Glucosidase (0.35 U/ml) and ρ‐NPG (1.5 mM ρ‐ nitrophenyl‐α‐D‐glucopyranoside) were dissolved in 0.1 M sodium phosphate buffer (pH 7.0), and 50 μl of each extract was used at 0.35 unit. The mixture was mixed with 100 μl of α‐glucosidase enzyme solution, preincubated at 37°C for 10 min, and then 50 μl of 1.5 mM ρ‐NPG was added at 37°C for 20 min. After stopping the reaction by adding 1 ml of 1 M Na_2_CO_3_, absorbance was measured at 405 nm using a UV spectrophotometer (Epoch microplate spectrophotometer, Biotek Instruments, Vermont, USA), and the inhibition rate (%) was calculated. Acarbose (Sigma‐Aldrich, St. Louis, MO, USA) was used as a positive control.

### 2.5 Reactive oxygen species inhibitory effects in HepG2 cells

The human liver cancer cell line (HepG2 human liver cancer cell line) used in this experiment was purchased from the Korea Cell Line Bank (Seoul, Korea). The cytotoxicity of the extract was measured using the MTT[3‐(4,5‐ dimethylthiazol‐2‐yl)‐2,5‐diphenyltetrazolium bromide] assay according to the method described by Ishiyama et al. (1996). reactive oxygen species (ROS) was measured using the fluorescent probe DCFH‐DA method. Cells were dispensed at a concentration of 5 × 10^4^ cells/well into a 96‐well plate (100 μl) and cultured at 37°C in a 5% CO_2_ incubator for 24 h. The extract was diluted with FBS‐free DMEM medium at a concentration of 100 μg/ml. The medium used for the culture was removed, and the diluted sample was added to the medium. After pretreating the sample for 24 h, 250 μM DCFH‐DA was added to each well for 1 h at 37°C. After washing the cells with PBS, 1 mM tert‐butyl hydroperoxide (TBHP) was added. The corresponding ROS between cells were measured using a fluorescent spectrophotometer (Perkin‐Elmer, Norwalk, CT, USA) for 120 min at an excitation wavelength of 485 nm and an emission wavelength of 530 nm.

### 2.6 Wound healing assay for APRE‐19 cell

ARPE‐19 cells were inoculated in a 24‐well plate at a concentration of 2 × 10^5^ cells/well. DMEM‐F12 (0% FBS) was used as the medium and a stabilization time of 7 h was set. The cells were observed under a microscope, and where the bottom of the 24‐well plate was not visible, scratch was created by scratching the bottom with a yellow tip. The existing culture solution was removed and replaced with a new culture solution (0% FBS in DMEM/F12). After the extract was treated with 10 μl per well so that the final concentration was 32 and 100 μg/ml, pictures of each well were taken after incubation at 37°C and 5% CO_2_ incubator. After scratching, the condition of each well was checked at 18 h and a picture was taken. After organizing the photographic data, the area of the scratch for each photograph taken was measured using the Image studio program, and the rate of wound healing was calculated.

### 2.7 Tyrosinase inhibitory activity

Tyrosinase inhibitory activity was measured according to the method described by Yagi et al. (1987). In the reaction zone, 0.2 ml of mushroom tyrosinase (110 U/ml) was added to 0.5 ml of 0.175 M sodium phosphate buffer (pH 6.8), 0.2 ml of 10 mM L‐DOPA, and 0.1 ml of sample solution and reacted at 37°C for 2 min. The DOPA chrome generated in the reaction solution was measured at a wavelength of 475 nm using a UV spectrophotometer (Epoch microplate spectrophotometer, Biotek Instruments, Vermont, USA). The tyrosinase inhibitory activity was expressed as the rate of decrease in absorbance of the sample solution (with and without addition).

$$\text{Inhibitory}\;\mathrm{activity}\left(\%\right)=\left(1-\text{absorbance}\;\mathrm{at}\;\text{the sample addition}/\text{absorbance}\;\mathrm{at}\;\mathrm{no}\;\text{addition}\right)\times 100$$

### 2.8 Statistical analysis

For statistical analysis, the mean and standard deviation of each measurement group was calculated using the SPSS statistical program (Statistical Package for the Social Science, Ver. 18.0, SPSS Inc., Chicago, IL, USA), and the difference between the mean of the measured values was independent. A sample *t*‐test (Student's *t*‐test) was performed, and a one‐way ANOVA was performed to determine whether there was a difference between treatment conditions.

## 3 RESULT AND DISCUSSION

### 3.1 ABTS and DPPH radical scavenging activity

The results of the analysis of ABTS and DPPH radical scavenging activity of sweet potato tip and tuber extracts are shown in Table 1. ABTS radical scavenging activity was in the range of 2.68–32.45 mg AAE/g, and was 32.45 mg AAE/g for *Pungwonmi* tips, which was the most active. The DPPH radical scavenging activity was in the range of 0.53–15.10 mg AAE/g, and was 15.10 mg AAE/g for *Pungwonmi* tips. Both ABTS and DPPH radical scavenging activity were higher in the tip than the tubers. The antioxidant activity of tuber was higher in *Sinjami* than that of *Juhwangmi* (*p* < .05), and the *Pungwonmi* tips was significantly higher than that of the other two cultivar (*p* < .05), and there was no significant difference between *Hayanmi* and *Juhwangmi* tips. According to a study by Lee, Park, et al. (2007), the high phenolic substances in sweet potato parts and their antioxidant activity corroborate the high content of phenolic substances and good antioxidant activity in leaves and tips in the present study. According to a study by Li et al. (2012), the ABTS radical scavenging activity of sweet potato leaves and stalks was in the range of 8.02–14.95 AAE/g, and the content of the leaves was higher than that of the stalks. In addition, according to a study by Woo et al. (2012), the ABTS and DPPH radical scavenging activity of sweet potato tubers were 4.38–6.63 and 5.46–9.58 mg/g, respectively, and are similar to the results of this study.

**TABLE 1:** ABTS and DPPH radical scavenging activity of different sweet potato cultivars.

Parts	Cultivar	ABTS radical scavenging activity (mg AAE/g)^1^	DPPH radical scavenging activity (mg AAE/g)^1^
Tips	*Hayanmi*	12.09 ± 1.13^b^ ^2^, ^3^	11.03 ± 39.34^b^
	*Pungwonmi*	32.45 ± 3.83^a^	15.10 ± 25.19^a^
	*Juhwangmi*	12.17 ± 5.14^b^	10.91 ± 33.75^b^
Tubers	*Sinjami*	10.49 ± 43.15^c^	6.76 ± 22.96^c^
	*Juhwangmi*	2.68 ± 5.72^d^	0.53 ± 7.43^d^

^1^ mg ascorbic acid equivalent (AAE) per dry weight g.

^2^ Each value expressed as the mean ± standard deviation (*n* = 3).

^3^ Different superscript in the same items indicate a significant difference by duncan's range test (*p <* .05).

### 3.2 ACE I inhibitory activity

**FIGURE 1:**
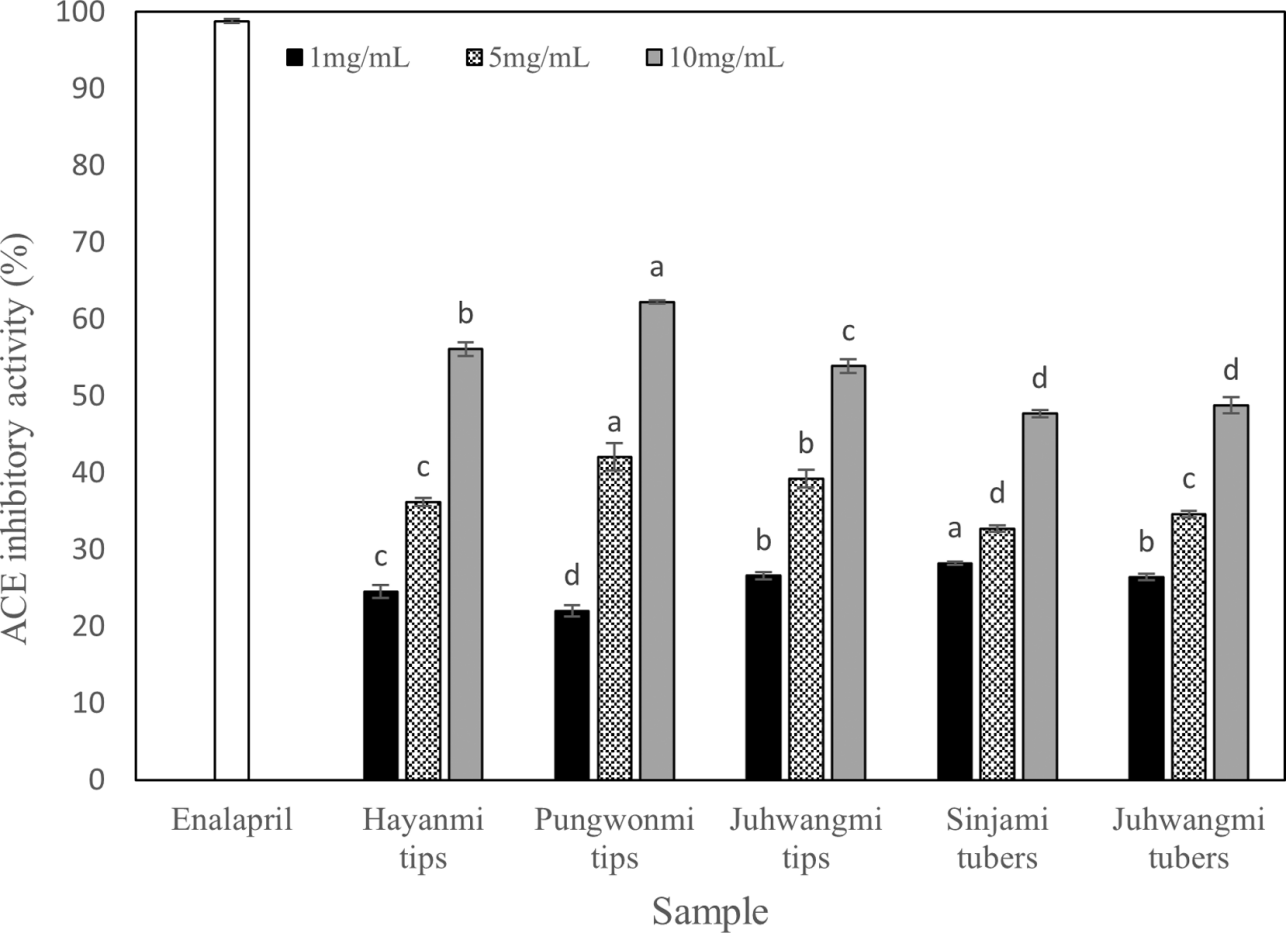
Angiotensin converting enzyme (ACE) inhibitory activity of different sweet potato cultivar. The concentration of positive control was 1 mg/ml. Data are expressed as mean ± SD. Different small letters in the same items indicate a significant difference among different cultivar by duncan's range test (*p* < .05).

The results of ACE inhibitory activity of sweet potato tip and tuber extracts are shown in Figure 1. ACE inhibitory activity significantly increased with increasing concentration in all cultivars, and showed 47.72%–62.25% activity at 10 mg/ml. ACE inhibitory activity was highest at the *Pungwonmi* tips and showed a high activity of 22.06% at 1 mg/ml, 42.05% at 5 mg/ml, and 62.25% at 10 mg/ml. In the study by Lee, Ahn, et al. (2007), which analyzed the antihypertensive activity of each sweet potato part, the ACE inhibitory activity was 1.5 times higher in the tips than in the tubers, showing a trend that is consistent with this study. As a result of statistical analysis of ACE inhibitory activity, there was a significant difference between tip and tuber at a concentration of 10 mg/ml (*p* < .05). In the tip, *Pungwonmi* cultivar was the highest, followed by *Juhwangmi* and *Hayanmi* cultivar (*p* < .05). However, there was no significant difference between cultivars in the tubers. In addition, a study by Lee, Ahn, et al. (2007), which analyzed the ACE inhibitory activity of sweet potato tips and tubers, showed excellent antihypertensive activity that was 1.9–3.7 times higher than that of perilla leaves, bean sprouts, and spinach. Enalapril, a treatment material for hypertension, was used as a positive control, and it showed 98.80% activity at 1 mg/ml. Studies showing the ACE inhibitory activity of herb plants, medicinal plants, and onion seasonings have been reported (Kwon et al., 2006; Lee et al., 2004).

### 3.3 α‐Glucosidase inhibitory activity

**FIGURE 2:**
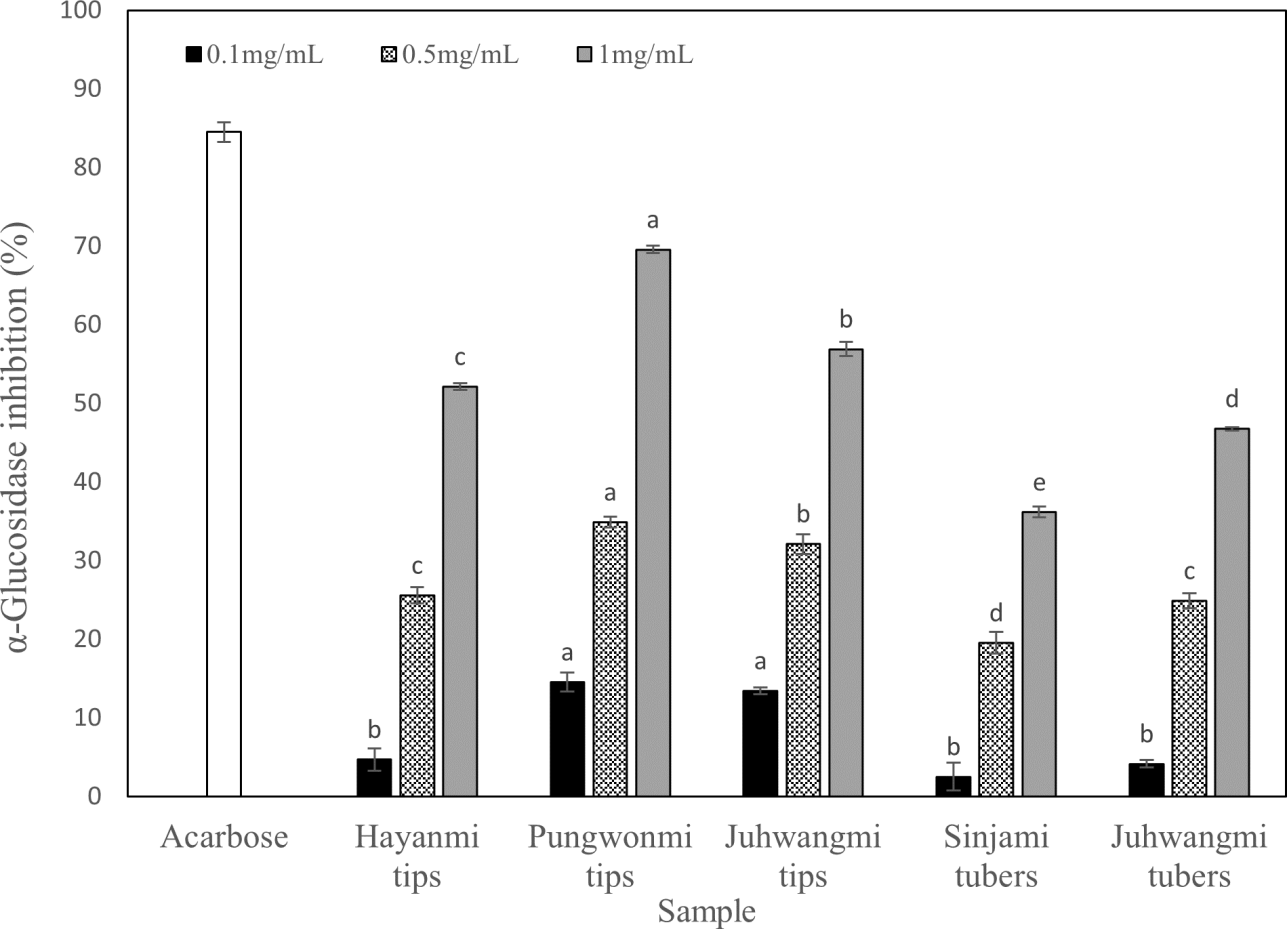
α‐Glucosidase inhibitory activity of different sweet potato cultivar. The concentration of positive control was 1 mg/ml. Data are expressed as mean ± SD. Different small letters in the same items indicate a significant difference among different cultivar by duncan's range test (*p* < .05).

The results of the antidiabetic activity of sweet potato tip and tuber extracts are shown in Figure 2. Antidiabetic activity significantly increased with increasing concentration of the extracts in all cultivars, and showed an inhibitory activity in the range of 36.17%–69.57% at 1 mg/ml. Acarbose, an antidiabetic treatment, which was used as a positive control in the experiment, showed 84.49% activity at 1 mg/ml. Antidiabetic activity was as high as 69.57% for *Pungwonmi* tips and 82% for acarbose. In a study by Matsui et al. (2004), it was reported that 3,4‐ dicaffeoylquinic acid, 3,5‐dicaffeoylquinic acid, and 4,5‐dicaffeoylquinic acid had excellent inhibitory effects on maltase and 3,4,5‐triCQA in diabetes model mice. When administered orally, the blood sugar content was significantly reduced.

### 3.4 ROS inhibitory effects in HepG2 cells

**FIGURE 3:**
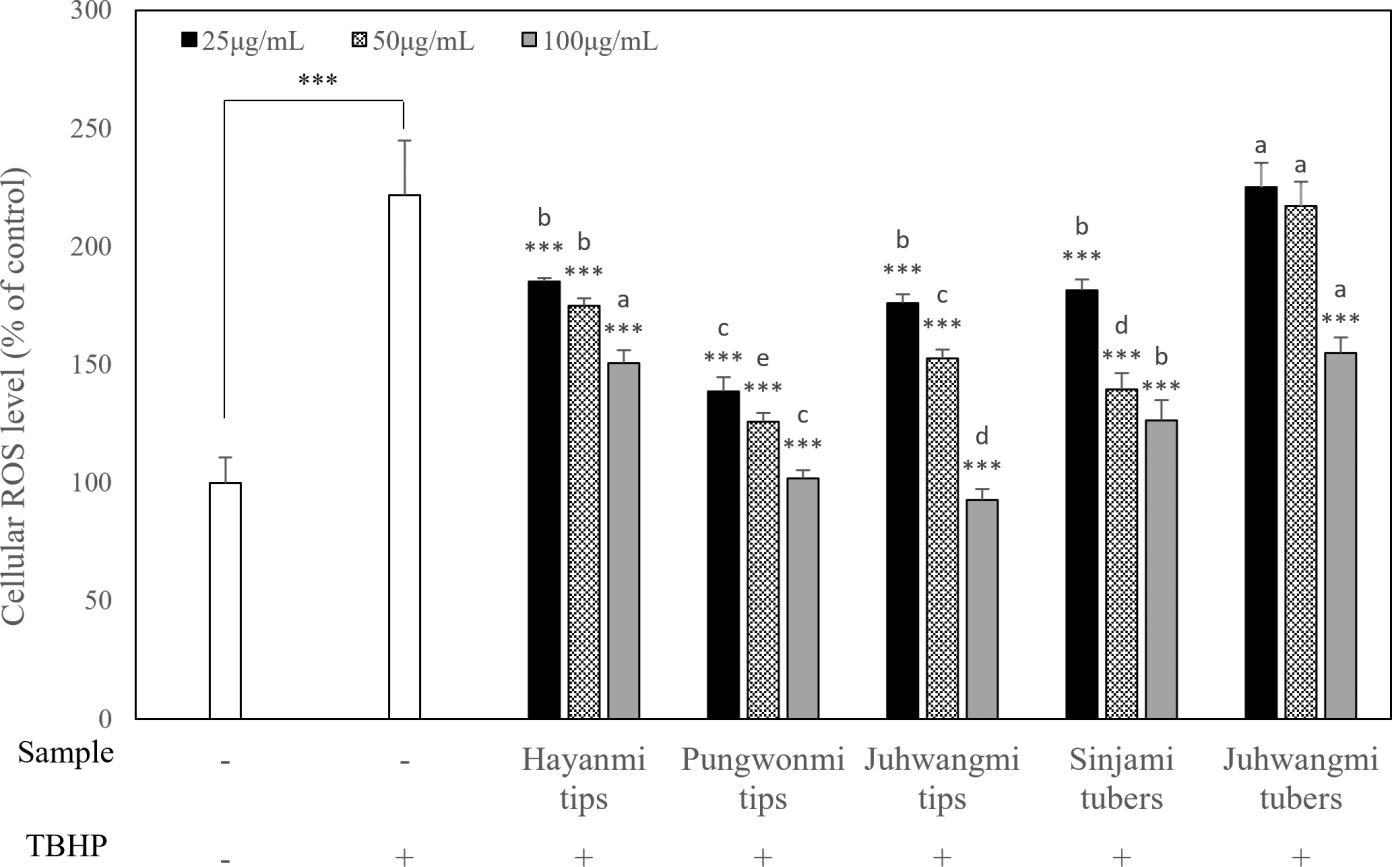
Effect of different sweet potato cultivar on the intracellular ROS formation induced by tert‐butyl hydroperoxide (TBHP). Data are expressed as mean ± SD. Different small letters in the same items indicate a significant difference among different cultivar by duncan's range test (*p* < .05). *p < .05, **p < .01, ***p < .001; significant difference compared to control.

The results of ROS inhibitory effects are shown in Figure 3. From the result of the ROS inhibition activity test using hepatocytes, ROS production increased in the cells induced oxidative stress with TBHP, compared to normal cells, however, the ROS production was inhibited in a concentration‐dependent manner in cells treated with sweet potato tips and tubers extracts. Particularly, it was found that *Pungwonmi* tips were effective in inhibiting ROS production. When oxidative stress was induced with TBHP in HepG2 cells, ROS production increased to 221.88% compared to that of the control, however, ROS production was suppressed by 25 and 50 μg/ml *Pungwonmi* tips to 138.70% and 125.94%, respectively. As a result of statistical analysis for ROS inhibitory effects in HepG2 cells, all samples except 25 and 50 μg/ml *Juhwangmi* tubers showed high significance (*p* < .05). Kwak et al. (2010) reported on the neuroprotective effect of purple sweet potato water extract against oxidative stress, and in a study by Jang et al. (2014), it was reported that the *Sinjami* cultivar had an excellent protective effect against cell damage caused by treatment of HepG2 cells with hydrogen peroxide (H_2_O_2_).

### 3.5 Wound healing assay for APRE‐19 cell

**FIGURE 4:**
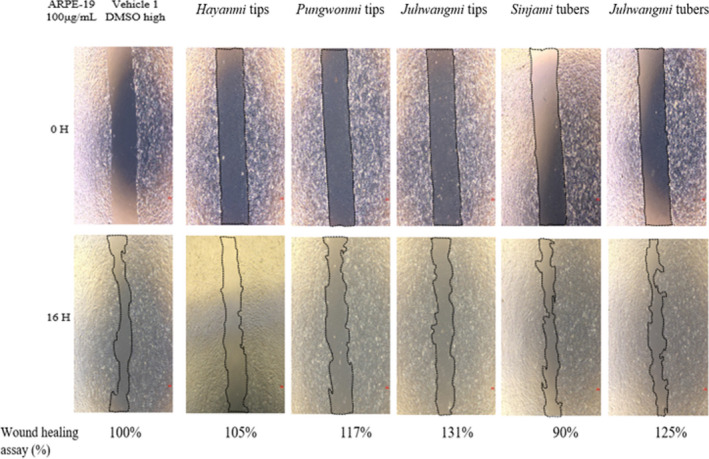
Wound healing image for ARPE‐19 cell from different sweet potato cultivars

The results of wound healing assay of sweet potato tip and tuber extracts are shown in Figure 4. Result of the Image J program analysis revealed that *Sinjami* tubers showed 75% wound healing effect in the 100 μg/ml treatment group. In the 32 μg/ml treatment group, *Sinjami* tubers showed 90% wound healing effect. *Sinjami* tubers are a representative of purple sweet potato cultivar with high anthocyanin content (Kim et al., 2012). Anthocyanins are reported to have antioxidant, antibacterial, hepatoprotective, and antihypertensive effects (Kang et al., 2003; Qi et al., 2005), and are known to be effective in treating eye disease (Kang et al., 2019). In a study by Kang et al. (2019), anthocyanin reduced the inflammatory response on the surface of the eye; they also reported its effect in improving the symptoms of dry eye syndrome, similar to the result of a study by Riva et al. (2017).

### 3.6 Tyrosinase inhibitory activity

**FIGURE 5:**
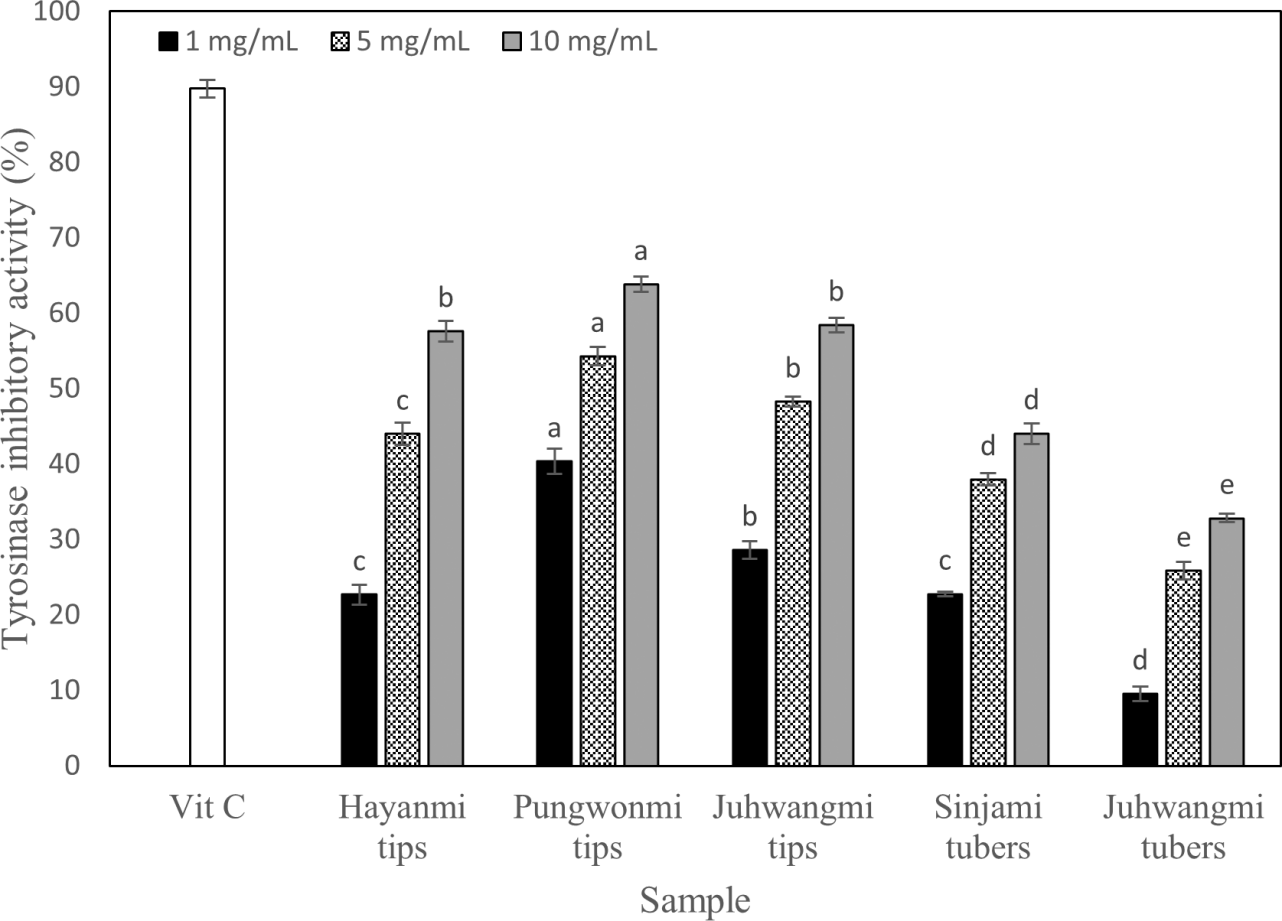
Tyrosinase inhibitory activity of different sweet potato cultivar. Data are expressed as mean ± SD. Different small letters in the same items indicate a significant difference among different cultivar by duncan's range test (*p* < .05).

The results of the tyrosinase inhibitory activity of sweet potato tips and tubers are shown in Figure 5. The positive control, vitamin C, showed an inhibitory rate of 89.76% at a concentration of 1 mg/ml, and the tyrosinase inhibitory activity of sweet potato tips and tubers was in the range of 32.82%–63.93% at 10 mg/ml. Among them, *Pungwonmi* tips showed the highest inhibitory activity (63.83% at 10 mg/ml). Statistical analysis confirmed that the tyrosinase inhibitory activity was a significant difference between tip and tuber. The tubers showed a significant difference between *Juhwangmi* and *Sinjami* cultivar (*p* < .05). The *Pungwonmi* tips showed significantly difference other cultivar, however, there was no difference between *Hayanmi* and *Juhwwangmi* (*p* < .05). Choi et al. (2011) studied the whitening activity of purple sweet potato extract, and the result showed 47.3% activity in *Sinjami* tubers, showing a trend similar to this study. The tyrosinase inhibitory activity of the tuber muscle was 32.82%–44.03%, and the tip was 57.59%–63.83%, indicating that the whitening effect of the tips was superior to that of the tubers. Lee et al. (2006) also confirmed the effect of tyrosinase inhibitory activity of sweet potato extract, and it was reported that the tyrosinase inhibitory activity increased as the concentration of the sweet potato extract increased.

## 4 CONCLUSION

This study evaluated the biological activities of antihypertension, antidiabetes, hepatoprotective, antioxidant, eye function, and skin whitening effects of sweet potato tips and tubers to develop the functional food and cosmetic materials. As a result of ABTS and DPPH radical scavenging activity, ‘*Pungwonmi*’ tips showed the highest activity. The results of ACE inhibitory activity for antihypertensive activity was also highest in ‘*Pungwonmi*’ tips. α‐Glucosidase and tyrosinase inhibitory activity increased significantly with increasing concentration in all cultivars; the activity was higher at the tips than the tubers. α‐Glucosidase inhibitory activity showed the highest activity of 69.57% at 10 mg/ml in ‘*Pungwonmi*’ tips. When oxidative stress was induced with TBHP in HepG2 cells, ROS production increased by 221.88% compared to that in the control, however the ‘*Pungwonmi*’ tips decreased by 102%. In the wound healing assay for ARPE‐19 cell experiment, the effect of creating a scratch on the ARPE‐19 cell was highest in the ‘*Sinjami*’ tubers. These results suggest that, sweet potato tips and tubers are excellent in improving eye disease, have whitening effects, and can be used as functional food and cosmetic material.

## FUNDING INFORMATION

Rural Development Administration (RDA), Republic of Korea, Grant number: PJ0142332020

## CONFLICT OF INTEREST

The authors declare that they do not have any conflict of interest.

## Data Availability

The data that support the findings of this study are available from the corresponding author upon reasonable request.
